# A High-Resolution Single Nucleotide Polymorphism Genetic Map of the Mouse Genome

**DOI:** 10.1371/journal.pbio.0040395

**Published:** 2006-11-14

**Authors:** Sagiv Shifman, Jordana Tzenova Bell, Richard R Copley, Martin S Taylor, Robert W Williams, Richard Mott, Jonathan Flint

**Affiliations:** 1 Wellcome Trust Centre for Human Genetics, University of Oxford, Oxford, United Kingdom; 2 Department of Anatomy and Neurobiology, University of Tennessee Health Science Center, Memphis, Tennessee, United States of America; Baylor College of Medicine, United States of America

## Abstract

High-resolution genetic maps are required for mapping complex traits and for the study of recombination. We report the highest density genetic map yet created for any organism, except humans. Using more than 10,000 single nucleotide polymorphisms evenly spaced across the mouse genome, we have constructed genetic maps for both outbred and inbred mice, and separately for males and females. Recombination rates are highly correlated in outbred and inbred mice, but show relatively low correlation between males and females. Differences between male and female recombination maps and the sequence features associated with recombination are strikingly similar to those observed in humans. Genetic maps are available from http://gscan.well.ox.ac.uk/#genetic_map and as supporting information to this publication.

## Abstract 2

A high-density SNP map based on outbred and inbred mice with male and female separation suggests a high degree of homology between mouse and human recombination.

## Introduction

The current genetic map of the mouse is based on data from crosses between inbred strains constructed using simple sequence length polymorphism markers in the late 1990s [1,2]. The most recent, and still the most comprehensive map, was created by backcrossing SPRET/EI *(Mus spretus)* to C57BL/6 and mapping the position of 3,368 markers on 982 progeny. The map consists of 2,302 genetically separated bins, with an average distance between bins of 0.61 centimorgans (cM) [3].

The completion of the mouse genome [4] together with the discovery of thousands of single nucleotide polymorphisms (SNPs) [5–7] has meant that high-resolution maps can be simply obtained by converting physical to genetic distances, since on average 1 cM corresponds to 1.6 megabases (Mb). However, although any number of SNPs can be combined to form a map using this approach [8], recombination rates are not constant across the genome [9,10], so at high resolution, the inferred genetic distances will be incorrect.

There are a number of applications for which accurate distances are needed. First, at high resolution, recombination-based genetic mapping of phenotypic variation will incur errors if incorrect genetic map distances are used. Recombination-based mapping is an efficient method to tackle a major challenge facing complex trait genetics: mapping quantitative trait loci (QTLs) into sufficiently small intervals to make gene identification possible. By exploiting historical recombinants that have accumulated over many generations, either in a genetically heterogeneous stock (HS) of mice descended from eight inbred progenitor strains [11,12], or in an advanced intercross [13], QTLs can be mapped into intervals of a few megabases. The accuracy of these methods depends on the accuracy of the genetic map. Additionally, as in human linkage mapping, a substantial difference in the number of informative male and female meioses will decrease power [14,15].

Second, accurate genetic maps are important for investigating recombination hotspots and the causes of recombination rate variation across the mammalian genome. For human populations, it has been possible to estimate fine-scale recombination rates from linkage-disequilibrium (LD) data, using a coalescent-based method and certain assumptions about population history. These data support the hypothesis of a two-stage process for recombination in which recombination rates are constrained over large scales, but rapidly change over a fine scale [10,16]. Consistent with this view, the location of hotspots rarely occurs in the same positions in humans and chimpanzees [17]. However, the features that induce and maintain recombination hotspots in mammals remain largely obscure. A high-resolution recombination-rate map in the mouse would make it possible to investigate these phenomena in a genetically tractable model organism.

We set out to construct a very high-resolution genetic map using more than 10,000 SNPs. One map was constructed using eight recombinant inbred (RI) lines (AXB, BXA, CXB, BXD, BXH, AKXD, LXS, and SWXJ). The second was constructed using recombinants that have accumulated over four generations in a genetically heterogeneous stock of outbred mice [18]. The HS are descended from eight inbred progenitors (AKR/J, A/J, BALB/cJ, CBA/J, C3H/HeJ, C57BL/6J, DBA/2J, and LP/J), and although their genomes are fine-grained mosaics of these founders, for the purpose of constructing the genetic map, we ignored their ancestry and focused only on the last four generations of the cross for which full pedigree information was available.

## Results

### Genotype Quality and Accuracy

A total of 13,367 SNPs were genotyped using the Illumina BeadArray platform (Illumina, San Diego, California, United States) with call rates of 99.86%. The average interval size between the selected SNPs based on National Center for Biotechnology Information (NCBI) build 34 is 167.0 kilobases (kb) (standard deviation [SD] = 141.5); 99.0% of the intervals are below 500 kb and 81.2% are below 250 kb. Genotype accuracy, based on the inheritance of alleles in 1,097 trios and 25 duplicate samples, is 99.99%. From 5,089 SNPs typed on 42 inbred strains by us and by Merck [19], there was 0.041% disagreement. Assuming an equal error rate in the two studies, the estimated accuracy of our data is 99.98%.

### Genetic Maps

Maps were created separately for HS mice and RI strains. Genotypes were obtained for 11,247 informative SNP markers in 2,293 HS animals. There are 84 HS pedigrees, eight of which are large, complex multi-generational families with inbreeding loops, encompassing the majority (61.1%) of individuals. The remaining 72 families are nuclear, with an average sibship size of 9.6 (range 2–34). The pedigrees consisted of 4,048 potentially informative meioses, of which the average number of informative meioses per marker was 1,296.7 (range: 9–2,755 per marker), and the average number of phase-known meioses was 198.1 (range: 7–964 per marker).

We estimated genetic distances using the software program CRIMAP [20]. Initially we took the physical order of marker from NCBI build 34 of the mouse genome and required each marker to exhibit linkage to at least one other marker on its chromosome. Because of the complexity of the HS pedigrees and the number of markers, it was not possible to include entire chromosomes in the analyses. Instead, each chromosome was analyzed using overlapping windows of five to 15 SNPs at a time, considering all possible orders within a given window of markers. Recombination rate estimates were also obtained using CRIMAP.

In three instances we obtained linkage support for physical order, but the estimated recombination rates indicated that unlinked markers were present in that SNP window. In the first instance, a SNP (mCV24244050) on Chromosome 5 appeared completely unlinked across the majority of families. The likely explanation is that the SNP is placed at the end of the genetic map (near the centromere), where recombination events are difficult to establish. In the other two cases, SNPs in the middle of chromosomes 8 (rs13479764) and 12 (rs13481579) appeared unlinked in the map. The number of informative meioses for these two SNPs was low, and an examination of the distribution of crossovers indicated that they occurred in just two families. On Chromosome 8, the inflated recombination rates were observed only in the maternal meioses, whereas on Chromosome 12, the inflation was observed in the male meioses. On Chromosome 12, the recombination rate was also inflated (but to a lesser extent) at a location eight markers upstream of the unlinked SNP. These inconsistencies may indicate the presence of chromosomal rearrangements. We excluded all these markers from the genetic maps.

Altogether we discarded 1,034 markers that mapped to multiple locations or did not map to any location on their assigned chromosome (see Materials and Methods). An additional 12 markers were discarded due to highly inflated recombination rates, which indicated inconsistencies in the map order. The remaining 10,202 markers had an average minor allele frequency of 0.26 (range: 0.003–0.5) and the average physical distance between consecutive markers is 248 kb (Table S1).

The total length of the sex-averaged genetic map is 1,630 cM. The average genetic inter-marker distance is 0.16 cM, the largest inter-marker interval is 6.15 cM (between 53.6 and 59.8 cM) on Chromosome 5, and the average genetic distance between unique map positions (map resolution) is 0.37 cM for the sex-averaged map (Table 1). There are 59 regions with inter-marker interval above 2 cM. Sex-specific maps were also obtained in the HS data and, as observed in other species, showed pronounced variation in the rate of recombination. The lengths of the female and male genetic maps are 1,817 cM and 1,386 cM, respectively (Tables S2 and S3).

**Table 1:**
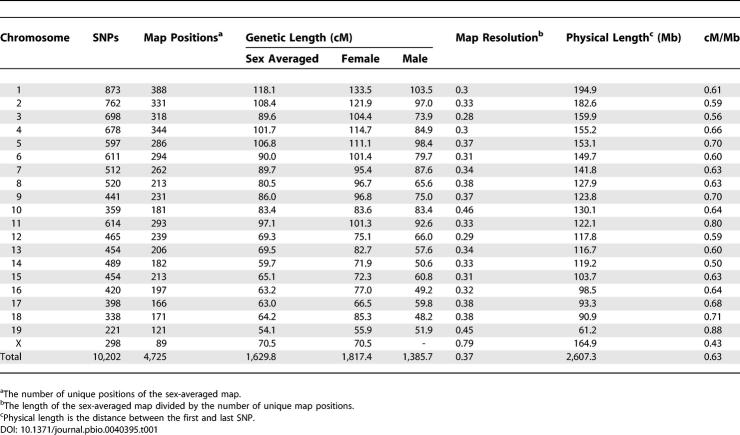
Description of the SNP Genetic Map in the HS Pedigrees

A consensus genetic map for recombinant inbreds was constructed using 11,609 informative SNPs and 267 strains from the eight sets of RIs. R/QTL was used to estimate the genetic distances between SNPs that were polymorphic in at least one of the RI sets [21]. On average, 52.6% of the 267 RI strains had informative genotypes per SNP (mean = 140 genotypes, SD = 60). Of the 273 RIs, six were not used for the map construction. AXB19, AXB20, AXB13, and BXA8 were excluded because their genotypes indicated that they could not be considered as independent strains [22,23]. AXB1 and AXB2 were excluded because of an extremely large number of flanking double recombination events (seven times more than other strains in the set). Twenty-three SNPs (0.2%) with three or more flanking double recombination events were excluded from the map construction. The genotypes of the rest of the SNPs are highly accurate: 124 genotypes (0.008% of genotypes) had an error log of odds (LOD) score of more than 3, which is consistent with the genotyping accuracy estimates of 99.99%. We also tested for segregation distortion from the expected 50% inheritance of each allele across all SNPs and RI strains. The combined *p*-value (Fisher method for combining *p*-values) across all strains, corrected for the number of SNPs, did not reach significance for any of the SNPs.

In the BXD set, 52 SNPs showed variation in genotypes that corresponded to the three different phases of development of the BXD RIs [24–26] (Table S4). Forty-seven SNPs are not polymorphic in the 26 BXD strains established from a single cross of a C57BL/6J female to a DBA/2J male, but are polymorphic in similar BXD strains established more than 20 y later. Five SNPs are not polymorphic in the first 36 BXD strains, but are polymorphic in the newest set of 53 BXD lines (BXD43–100). The 52 SNPs are not polymorphic in other inbred strains examined, including highly related strains such as DBA/1J and C57BL/10J. These data indicate that the 52 SNPs are recent mutations that have arisen since the creation of the BXD lines. Twenty-four of the mutations are in the DBA/2J strain and 28 are in C57BL/6J. For the map purposes, we treated the BXD as three different sets for the analysis of the 52 SNPs. The genotypes for the non-polymorphic subset were treated as missing values.

We converted RI recombination frequencies to the distances expected for a single-generation meiotic map. The total genetic length of the RI map from proximal to distal SNP on each chromosome is 1,605 cM (Table S5). The genetic lengths of each chromosome are presented in Table 2, and the number of recombinants in each set is shown in Table 3. One BXD subset was developed using advanced intercross populations [26] and therefore contains approximately 1.7 times more recombinants per line. The average number of recombinations in each of the other sets is correlated with the number of polymorphic SNPs in the set (Spearman correlation coefficient *r* = 0.84, *p* = 0.02). The average distance between recombination events (map length divided by the number of recombinations) in the RI map is 0.11 cM (29.3 cM per strain) or 177.1 kb (47.3 Mb per strain). The average genetic distance between SNPs in the RI map is 0.14 cM or 216.4 kb, and the average resolution is 0.45 cM (Table 2).

**Table 2:**
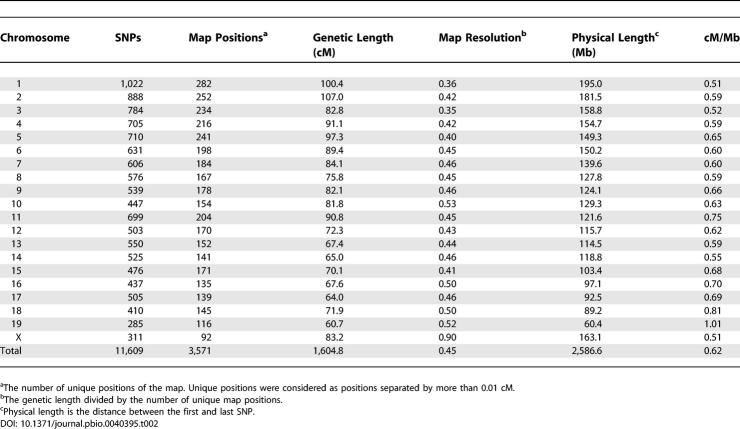
Description of the SNP Genetic Map in the RIs

**Table 3:**
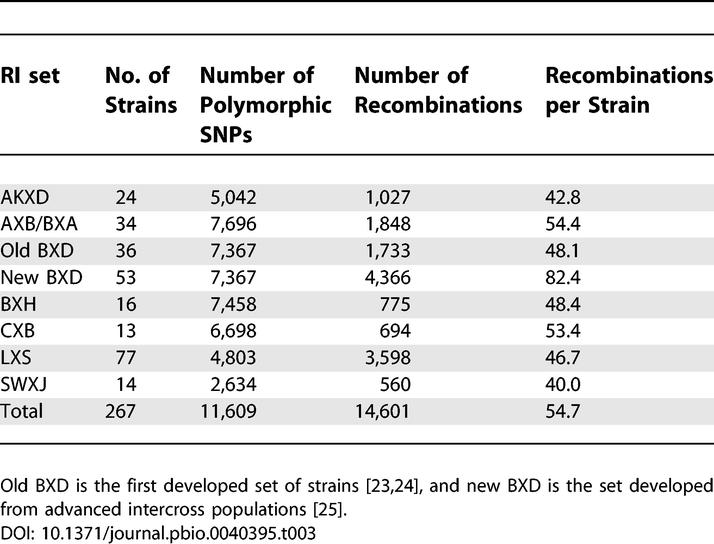
Average Number of Recombinations for Different RI Sets

### Recombination Rates

We compared genetic and physical distances and calculated recombination rates, expressed as centimorgans per megabase across the genome. The genome-wide average recombination rate is 0.63 cM/Mb for the HS sex-averaged map and 0.62 cM/Mb for the RI map. The rates varied between chromosomes and range from 0.43–0.88 and 0.51–1.01 cM/Mb per chromosome for the HS and RIs, respectively.

Variation in recombination rates between chromosomes is partly accounted for by chromosome size (HS: *r* = −0.60, proportion of variance explained R^2^ = 0.35, *p* = 0.0056; RIs: *r* = −0.85, R^2^ = 0.73, *p* = 1.6 × 10^−6^). There is a negative correlation between chromosome size and chromosome average recombination rate, with the recombination rate of the smallest chromosome (Chromosome 19) being approximately 1.5-fold higher than that of the longest chromosome (Chromosome 1) in the HS and 2-fold higher in the RIs. The correlation between chromosome size and recombination rate is higher in female HS (*r =* −0.61, *p* = 0.0041) than in male HS maps (*r =* −0.47, *p* = 0.044). The X chromosome does not account for this difference between females and males because it exhibits a lower rate of recombination relative to the rate expected for its size. The correlation between chromosome size and recombination rate in females excluding chromosome X is nearly the same (*r =* −0.61, *p* = 0.0054).

**Figure 1:**
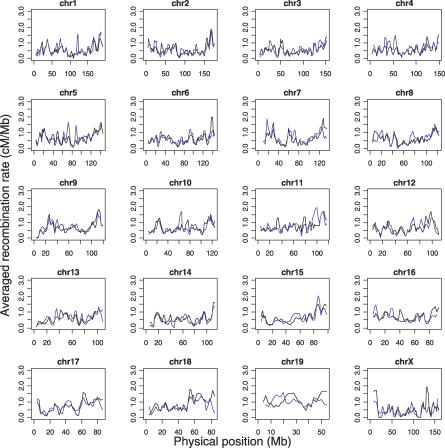
Average Recombination Rates for All Chromosomes Rates for RIs are shown as a black line and for HS as a blue line. The ratio between the genetic distance and physical distance was calculated using a sliding window of 5 Mb and a shift of 2 Mb between windows centers, assuming a constant rate of recombination between two adjacent markers.

We calculated recombination rates along chromosomes using non-overlapping windows of 1, 3, and 5 Mb. These window sizes were chosen as a compromise between the limits of the marker resolution (about 200 kb) and the need to sample sufficient recombination events within a window. The correlation between recombination rates calculated for each window for the HS and RI maps is 0.50, 0.60, and 0.66, respectively (*p* < 1 × 10^−60^ for all windows) (Figure 1). The range of recombination rates for 1-, 3-, and 5-Mb windows varies from 0–6, 0–3, and 0–2 cM/Mb, respectively, with higher rates of recombination near the telomeres.

**Figure 2:**
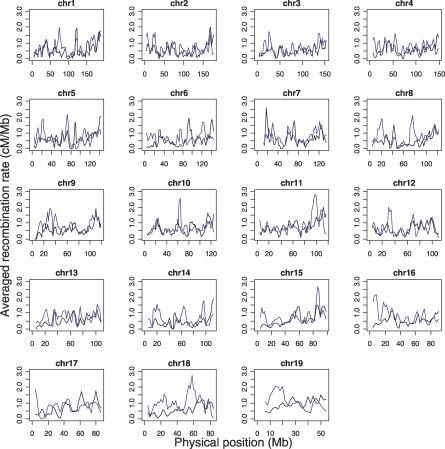
Sex-Specific Recombination Rates in the HS Average recombination rate for all chromosomes calculated similar to the HS sex-averaged map, for males (black line) and females (blue line).

We calculated recombination rates in males and females separately using the sex-specific HS map for all autosomes. On average, the autosomal rate of recombination is higher in females (0.72 cM/Mb) than in males (0.57 cM/Mb). The recombination rate in females is notably higher near the centromere, whereas in males it is higher near the telomere (a highly significant difference: *p* = 2.3 × 10^−7^), similar to the pattern observed in humans. We also examined the correlation between male and female recombination rates and identified many regions showing high recombination rate in one sex, but not the other (Figure 2). For example, there are four sex-specific peaks on Chromosome 6 between 60 Mb and the end of the chromosome. The correlation between sex-specific recombination rates calculated using 1-, 3-, and 5-Mb non-overlapping windows for males and females are 0.28, 0.32, and 0.33, respectively (*p* < 1 × 10^−6^).

### Correlation of Recombination with Sequence Parameters

To investigate the association between recombination and sequence features, we analyzed megabase-sized regions with extreme rates of recombination in both HS and RIs. A 1-Mb non-overlapping window was used to identify the top and bottom 10% of intervals with the highest and lowest recombination rates (termed here recombination “jungles” and “deserts,” respectively). Out of the 494 extreme regions, 154 are recombination jungles and deserts in both HS and RIs (84 recombination jungles and 70 recombination deserts). Thirteen regions are discordant, being in the high 10% based on one map (in ten of 13 cases in the HS map) and in the low 10% based on the other map.

**Figure 3:**
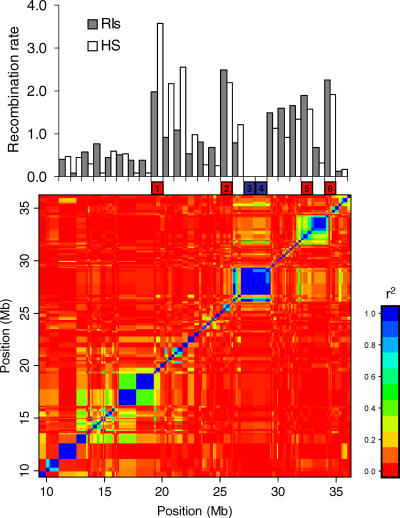
Relation between LD Levels in Inbred Strains and Recombination Rates in HS and RIs on Chromosome 7 The top figure is the recombination rate (cM/Mb) for the HS and RIs in 1-Mb non-overlapping windows across a 25-Mb region in Chromosome 7. Below, the six boxes represent the location of four recombination jungles (in red) and two deserts (in blue) of 1 Mb each. The bottom figure is the LD structure of the same region based on the SNP genotypes of 55 inbred strains. Each dot represents the pairwise LD level between two different SNPs. Regions of high LD measured by *r^2^* are in blue, whereas regions of linkage equilibrium are in red. As can be seen in this example, deserts tend to overlap with large blocks of LD, and jungles are typically located on the border of the blocks. The overlap proportion of the four recombination jungles with haplotype blocks is 0.00, 0.58, 0.55, and 0.35 for regions 1, 2, 5, and 6. The two deserts (3 and 4) overlap completely with haplotype blocks.

To test whether jungles and deserts are conserved, we defined the haplotype block structure of the mouse genome using 55 inbred strains that were genotyped for the same SNP panel. As in human populations, haplotype blocks were defined to be regions where there has been little historical recombination [27]. We investigated the relations between recombination rates and LD structure by comparing the position of jungles and deserts with the boundaries of haplotype blocks (Figure 3). We identified 682 haplotype blocks with an average size of 0.90 Mb (range: 0.0086–10.23 Mb), spanning a total of 611.56 Mb (23% of the genome). To compare block boundaries with the recombination jungles and deserts regions, we aligned the blocks with the jungles and deserts, and for each 1-Mb region, examined the proportion within haplotype blocks. An overlap of more than 80% between the haplotype blocks boundaries and a jungle or a desert was considered a region that predominantly lies within haplotype blocks. Fifty-nine percent of the total span of all recombination deserts overlap with haplotype blocks compared to only 12% for the jungles (*p* = 2.4 × 10^−13^). Only one jungle predominantly lies within a haplotype block in contrast to 33 (47.1%) of the deserts. Nevertheless, there are 19 (25.7%) deserts that do not correspond to haplotype blocks.

To search for factors that predict recombination jungles and deserts, we first examined the association between recombination rate and global sequence features. We found differences in the abundance of many sequence variables, including simple repeats, interspersed repetitive sequences, and transposable elements (Table 4), but gene and SNP density were not significantly enriched in jungles or deserts. We found a higher abundance of simple repeats in recombination jungles, including (CA)_n_ repeats (relative risk [RR] = frequency in jungles/frequency in deserts = 1.8), (TG)_n_ repeats (RR = 1.9), and low-complexity repeats (RR = 1.4). In contrast, (TA)_n_ repeats (RR = 0.79) and some long interspersed nuclear elements (LINEs) (RR = 0.6) are underrepresented in recombination jungles, as seen also in the study of human recombination hotspots [16]. An individual LINE repeat (termed L1_MM), enriched in recombination deserts, is the most significant feature associated with recombination (RR = 0.3, *p* = 2.8 × 10^−35^).

**Table 4:**
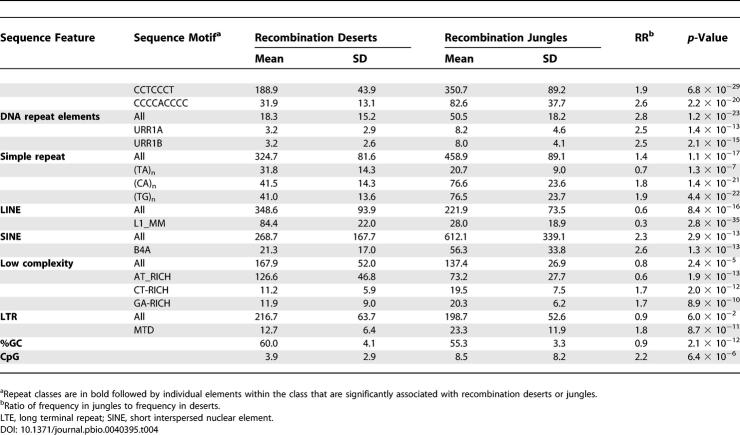
Repeat Elements and Sequence Variables Associated with Recombination Jungles and Deserts

**Figure 4:**
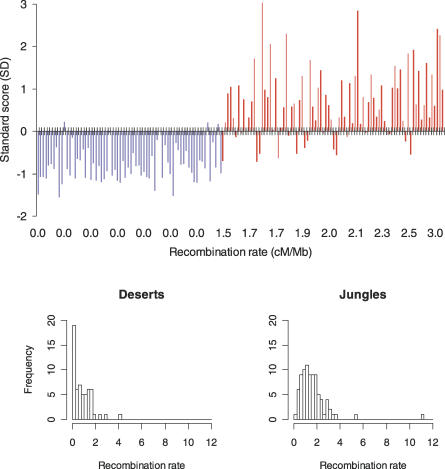
Recombination Jungles and Deserts Enrichment of CCTCCCT motif in recombination jungles (upper panel). The standard deviation (SD) from the mean number of CCTCCCT motifs is plotted against recombination rate. Red bars are 1-Mb regions included in the top 10% recombination rate in both HS and RIs (recombination jungles), and blue bars are regions in the bottom 10% (recombination deserts). Histograms of human recombination rates for the orthologous regions of the mouse recombination jungles and deserts (lower panel).

We examined the seven nucleotide sequence motif (CCTCCCT) previously shown to be enriched in human recombination hotspots [16]. We compared the number of occurrences of this sequence in recombination jungles and deserts. The CCTCCCT motif is significantly enriched in recombination jungles (RR = 1.9, *p* = 6.8 × 10^−29^), and the number of motifs is a very strong predictor of recombination jungles versus deserts (Figure 4A): it explains 63% of the deviance in a logistic regression model. In addition to CCTCCCT, we also tested a nine nucleotide oligomer (CCCCACCCC) that was also identified in human recombination hotspots. The CCCCACCCC motif is also enriched in recombination jungles (RR = 2.2, *p* = 2.2 × 10^−20^), but it does not significantly add to the deviance explained in a logistic regression model. Looking at recombination rate across the whole genome, the correlation between the CCTCCCT motif and the average recombination rate in non-overlapping windows of 1 Mb is 0.35 (*p =* 8.8 × 10^−71^).

We tested for the effect of sex on the association of the CCTCCCT motif with recombination. We used the HS sex-specific recombination rates calculated in larger windows (2 Mb) to make the estimates comparable to those obtained from sex-averaged estimates. The CCTCCCT motif was found to be associated with recombination rates in both males and females, with correlation of 0.35 and 0.26, respectively (males, *p* = 1.5 × 10^−35^; females, *p* = 1.1 × 10^−18^). We did not find associations between other sequence elements and recombination rates that differed between the sexes.

Since our findings suggest that the role of the CCTCCCT motif in recombination is conserved across different mammalian species, we asked whether the position of the motif is conserved between species. We identified the orthologous regions corresponding to the 154 regions of mouse jungles and deserts in the genomes of the rat, dog, chimpanzee, and human. The density of the CCTCCCT motif (counts per Mb) is highly correlated between all species across the different regions (correlation between mouse and rat: 0.84, dog: 0.67, chimpanzee: 0.67, and human: 0.74, maximum observed *p* = 5.5 × 10^−20^). To see how these cross-species correlations in the frequency of the CCTCCCT motif correspond to recombination rates, we calculated the recombination rate (cM/Mb) for the mouse orthologous regions in humans using the Rutgers high-resolution genetic map. Recombination rates in humans are on average higher in regions orthologous to mouse jungles compared to regions orthologous to deserts (*p* = 1.6 × 10^−5^), and correlate significantly with the mouse rates: the correlations are 0.35 (*p* = 2.0 × 10^−5^) and 0.42 (*p* = 2.2 × 10^−7^) with the values in RIs and HS, respectively. Figure 4B plots the frequency of recombination rates for the human regions that are orthologous to mouse jungles and deserts.

## Discussion

We have constructed one of the most dense and accurate genetic maps for any organism. Using both outbred animals and recombinant inbred lines, we genetically placed more than 10,000 markers onto the mouse genome, yielding an average genetic inter-marker distance of 0.15 cM. The average resolution is 0.37–0.45 cM/Mb for the sex-averaged maps. Even small numbers of genotype errors will inflate genetic map distances, and this factor has often made it difficult to draw conclusions about the factors that influence local recombination rates. Our high level of genotyping accuracy (~99.99%), the use of large pedigrees, and the construction of separate maps based on two types of populations has result in a highly reliable linkage map and highly accurate estimates of recombination rates.

Our map was constructed using HS mice that were also employed to find QTLs [11]. We collected 101 phenotypes for 1,904 HS mice (389 parents of these animals were also genotyped but not phenotyped, accounting for the 2,293 HS animals used in map construction). For the QTL analysis, 10,202 markers unambiguously positioned on the HS were used, supplemented by a further 1,910 informative markers whose locations were derived by interpolation from the physical map then available (NCBI build 34). Thus a sex-averaged map of 12,112 markers was used for QTL identification [11]. We have not yet explored the effect on QTL identification of using sex-specific maps.

We found that chromosome-specific recombination rates do not greatly differ between outbred (HS) and inbred populations (RIs): there are very few regions with discrepant recombination rates. Petkov et al. [28] suggest that inbreeding results in strong selection for specific allele combinations in mouse inbred lines: selection against recombination occurs when a combination of alleles at linked loci confers a survival advantage during the inbreeding process. Our data do not support the hypothesis of lower recombination events in inbreds or of strong selection forces that favor specific alleles. The inheritance of alleles in the RIs is not significantly different from what is expected.

We do see, however, some differences related to chromosome size. Recombination in the RIs appears to be more constrained by chromosome size, which explains 73% of the variance in the chromosome average recombination rate, whereas in the HS, it explains only 35% of the variance. Higher recombination rates are observed in the HS relative to the RIs in the large chromosomes, and lower rates in the small chromosome (with the exception of the X chromosome). We cannot at this point explain this observation.

We found remarkable similarity in the features that contribute to variation in recombination rates in mouse and human chromosomes. We confirmed that chromosome, position on the chromosome, sex, and sequence composition are common important factors. In both species, differences in recombination rates are correlated with chromosome size (or arm size) and proportional distance from the centromere. It is possible that, as has been suggested in humans, variation in recombination rates between chromosomes occurs because each chromosome arm is constrained to have a single chiasma, and the effect of fixing the lower limit of recombination will affect smaller chromosome arms more than larger [29,30]. Unlike human chromosomes, mouse chromosomes are all acrocentric (they lack a fully-formed P arm). Humans therefore have approximately twice as many chromosomal arms as mice, which would explain the difference in average recombination rates: our estimate of 0.6 cM/Mb is about half that found in humans. However, data from rats do not support this notion [31].

In both humans and mice, the average recombination rate in females is higher than males. Recombination rates are higher near the centromeres for females and towards the telomeres for males [32,33]. The correlation between female and male recombination rates across the whole genome is relatively weak, with many sex-specific peaks and troughs in recombination rates. This suggests that a larger proportion of the variance in recombination can be explained by sex-specific recombination events. A direct measure of recombination using immunohistological methods has shown that sex, and not genotype, influences recombination [34].

The main difference between the LD landscapes of the human and mouse genome is the presence of large haplotype blocks of up to several megabases in size in mouse inbred strains. We have shown that the position of these haplotype blocks tends to coincide with recombination deserts. In contrast, recombination jungles coincide with borders of these blocks or regions without evident block structure. An important implication of this observation is that genetic variants (such as those underlying quantitative trait loci) that lie within a recombination desert will be difficult to identify with the SNP maps we have generated.

We exploited the LD landscape of the mouse genome to concentrate on regions of very high-recombination rates (jungles) and very low rates (deserts). We used the selected jungles and deserts, as well as the recombination rates in the whole genome, to study sequence features that were shown to influence global and local recombination rates in humans. A major limitation of this analysis is that the location of recombination hotspots within jungles is unknown. Although deserts are essentially homogenous regions without recombination, jungles are presumably heterogeneous regions with an uneven distribution of recombination events. Therefore, in this study we focused on factors that are known to influence recombination in humans.

In humans, sequence motifs are an important determinant of variation in recombination rate. Analyses of the sequence correlates of recombination based on low resolution genetic maps have been unable to identify motifs that independently predict recombination rate. Most of the correlations have low coefficients and the sequence variables are correlated with each other. High resolution mapping of recombination hotspots in humans found CCTCCCT oligomers to be the strongest signal of recombination hotspots [16]. The motif is also the strongest predictor of recombination jungles in the mouse, explaining 63% of the deviance. In humans, this motif is found within the long terminal repeats of two retrovirus-like transposons, THE1A and B. The mouse genome does not contain this motif, providing additional support for its independent effect.

Our data support a two-stage model of recombination in which recombination rates are constrained over large scales, but are rapidly evolving on a small scale. The conservation of recombination deserts in inbred lines, shown by haplotype blocks and the presence of some of those deserts also in humans, are also consistent with this model. Comparisons between species using low-resolution sex-averaged maps have previously detected only a slight positive correlation between recombination rates [31]. The fact that the frequency of the CCTCCCT motif is well correlated across species in the regions that we studied, whereas the location of hotspots is not conserved, suggests that an interaction of several elements, including the CCTCCCT motif, influences recombination. The high degree of similarity between the factors that are correlated with mouse and human recombination rates, together with the correlation in recombination rates itself, implies the existence of a common mechanism that influences recombination rates in mammalian genomes. The genetic maps we have made are available from http://gscan.well.ox.ac.uk/#genetic_map and as Tables S1–S5.

## Materials and Methods

### SNP selection and genotyping.

We selected SNPs across the genome that distinguish between the eight HS founders (A/J, AKR/J, BALB/cJ, DBA/2J, C57BL/6J, LP/J, I, and RIIIS/J). We used datasets to select SNPs that are validated and polymorphic in at least some of the HS founders [6,19,35]. SNPs were chosen on the basis of their position in the genome, regardless of any known, or putative, function. SNPs were merged together and remapped onto the mouse genome (NCBI build 34). SNPs closer than 50 kb with identical strain distribution patterns (SDP) were discarded. All gaps without SNPs over 500 kb were filled using SNPs from Celera, Affymetrix SNPs, and from a comparative study of CZECHII/Ei. All genotyping was carried out at Illumina (San Diego, California, United States) using the BeadArray genotyping platform. PEDSTATS [36] and PedCheck [37] were used to identify non-Mendelian transmissions in the HS data. The Wellcome–CTC mouse strain SNP genotypes are available at http://www.well.ox.ac.uk/mouse/INBREDS.

### Genetic map construction: HS.

The mapping algorithm used the physical sequence positions to determine the initial order of the markers on the map, followed by estimation of linkage support for the physical order, and maximum-likelihood estimates of the recombination fractions. The majority of markers were present in NCBI build 34 of the mouse genome, and where these were unavailable (125 SNPs), build 33 locations were used to interpolate marker order on build 34. We used CRIMAP [20] to construct linkage maps applying the Kosambi map function [38].

Linkage support for physical order was obtained in two stages. The first step was to confirm localization of each marker to the chromosome assigned by its reported physical location on NCBI build 34 of the mouse genome. In the set of 11,247 informative markers, we required each marker to exhibit linkage to at least one other marker on that chromosome, with odds greater than 1000:1, using the *twopoint* option in CRIMAP. Altogether, 11,199 markers were linked to at least one marker on the chromosome with a LOD > 3, and 48 markers were discarded at this stage. The second test of map order consisted of the removal and remapping of each marker onto the map. A marker was kept in its physical NCBI build 34 location only if the likelihood of the physical location was within 1 LOD unit of the highest likelihood obtained.

Because of the complexity of some of the pedigrees and the number of markers, it was not possible to include entire chromosomes in the analyses. Therefore, each chromosome was analyzed using overlapping windows of five to 15 SNPs at a time, by considering all possible orders within a given window of markers using the *all* function in CRIMAP. If a marker did not map to within 1 LOD unit of its physical location, we searched for the highest likelihood of placement along the remaining windows for that chromosome. At this stage, 10,213 markers mapped to their physical location using this method. The remaining 986 markers mapped to multiple locations along the designated chromosome.

Once linkage support for marker order was established, recombination rate estimates were obtained for the 10,213 markers in CRIMAP. Recombination rate estimates were calculated using the *fixed* function of CRIMAP in a series of overlapping windows of five to 15 SNPs using the maximum number of estimated data points (over all windows) to calculate the recombination fraction between each pair of markers. Following recombination rate estimation, two markers in the map had inflated recombination rates and appeared completely unlinked in all windows analyzed. Because the density of markers indicated that these were highly unlikely events, we excluded these markers from the maps, and excluded one window on Chromosome 12 for which two such events occurred within a window of nine consecutive markers. As a result, 10,202 markers were placed on the map.

In the final set of 10,202 markers, we estimated double recombinant events, which may indicate erroneous genotypes or potential chromosomal rearrangements. We estimate the rate at which two or more recombinant events occurred within non-overlapping windows of five markers using the *chrompic* function in CRIMAP. The resulting estimate over the genome was 2.3 × 10^−4^, taking into account the average number of informative meioses per chromosome. However, we did not exclude any genotypes at this stage on the premise that the double recombinant events may represent chromosomal rearrangements, in particular, deletions.

### Genetic map construction: RIs.

The RI sets include: AXB (22 individuals, 7,696 polymorphic SNPs), BXA (18 individuals, 7,696 polymorphic SNPs), CXB (13 individuals, 6,698 polymorphic SNPs), BXD (89 individuals, 7,367 polymorphic SNPs), BXH (16 individuals, 7,458 polymorphic SNPs), AKXD (24 individuals, 5,042 polymorphic SNPs), LXS (77 individuals, 4,803 polymorphic SNPs), and SWXJ (14 individuals, 2,634 polymorphic SNPs).

Within each set of RI animals, we deleted any non-polymorphic SNPs from each set, as well as SNPs whose flanking sequences were not mapped to a single location in the NCBI build 34 mouse genome assembly. The genotypes were recorded as 1 or 2, based on the alleles of the founders, and heterozygotes were treated as missing values. The SNPs were ordered based on build 34. The number of double recombinations of adjacent SNPs was calculated for each SNP and each strain. If more than two double recombinants were found for a SNP interval, it was taken to indicate potential genotype errors or errors in the order of markers. Markers with more than two double recombinations were excluded. For the genetic distance estimation, the genotypes of the eight RIs sets from 267 strains were combined into one dataset. Alleles of SNPs that are not polymorphic in an RI set were treated as missing data. The error LOD score [39] was calculated for each genotype to identify possible genotyping errors using the R/QTL [21] function *calc.errorlod*. R/QTL was used to estimate the genetic distances between 11,609 SNPs that were polymorphic in at least one of the RI sets.

### Haplotype blocks.

Inbred strains were genotyped for the same panel of SNPs with the Illumina platform as mentioned above. Haplotype blocks were defined using a 95% confidence bound on D′ (the confidence interval algorithm) using Haploview 3.0. The following 55 inbred strains were selected from 72 inbred strains after removing 17 strains that are in high correlation (>0.8) with other strains: 129S6/SvEv, 129X1/SvImJ, A/J, AKR/J, ALR/LtJ, ALS/LtJ, BALB/cByJ, BPH/2J, BPL/1J, BPN/3J, BTBR T+ tf/J, BUB/BnJ, C3HeB/FeJ, C57BL/10J, C57L/J, C58/J, CALB/RkJ, CBA/J, CE/J, DBA/1J, DDY/JclSeyFrk, EL/SuzSeyFrk, FVB/NJ, I/LnJ, ILS, IS/CamRkJ, ISS, KK/HlJ, LEWES/EiJ, LG/J, LP/J, MA/MyJ, NON/LtJ, NOR/LtJ, NZB/BlNJ, NZO, NZW/LacJ, PERA/EiJ, PERC/EiJ, PL/J, RBA/DnJ, RBF/DnJ, RF/J, RIIIS/J, SB/LeJ, SEA/GnJ, SENCARA/PtJ, SF/CamEiJ, SJL/J, SM/J, ST/bJ, SWR/J, WMP/PasDnJ, WSB/Ei, and YBR/Ei.

### Sequence features.

We obtained gene and repeat features from the Ensembl database (data derived from mouse assembly NCBI build 34) using standard Perl modules [40]. GC content and the presence of particular sequence motifs were obtained by directly scanning DNA sequences of interest using bespoke Perl scripts. SNP density was calculated based on the SNPs from the Broad Institute (http://www.broad.mit.edu/snp/mouse/) together with SNPs from Perlegen (http://mouse.perlegen.com/mouse/download.html). We used the Student *t*-test to compare the abundance of features in recombination jungles and deserts. Logistic regression modeling was performed using the glm function in the R statistical analysis package version 2.1.1, with the type of region (jungle vs. desert) as the response, and the frequency of the sequence motif in the region as explanatory variable. Goodness of fit was tested using the model's deviance (twice the log likelihood ratio). Orthologous regions were defined between mouse (Build34 mm6, University of California Santa Cruz Genome Browser [http://genome.ucsc.edu/] nomenclature) and other assembled genomes (human, hg17; rat, rn3; dog, canFam1; and chimpanzee, panTro1) using the Genome Browser “Net” and “Chain” alignments as described by Taylor et al. [41].

## Supporting Information

Table S1 A High-Resolution Genetic Map of the Mouse Genome The map gives the sex-averaged genetic position of 9,904 SNP markers from 2,293 heterogeneous stock mice. The columns are the marker name (marker), the genome build of the markers' physical position (genome_build), the chromosome, base pair (bp) position, and genetic position (cM).
(830 KB XLS)

Table S2 Male-Specific Genetic Map of the Mouse Genome Based on HS Pedigrees The columns are the marker name (marker), the genome build of the markers' physical position (genome_build), the chromosome, base pair (bp) position, and genetic position (cM).
(830 KB XLS)

Table S3 Female-Specific Genetic Map of the Mouse Genome Based on HS Pedigrees The columns are the marker name (marker), the genome build of the markers' physical position (genome_build), the chromosome, base pair (bp) position, and genetic position (cM).
(976 KB XLS)

Table S4 SNPs That Distinguish Subsets of BXD Strains The columns are the marker name (marker), the chromosome, base pair (bp) position, the genotype of the BXD parental strains C57BL/6J (B) and DBA/2J (D), the genotypes of two highly related strains C57BL/10J and DBA/1J, the genotypes of the BXD lines sorted on the basis of the three different phases of development. B indicates an allele from C57BL/6J, D indicates alleles from DBA/2J, and H is heterozygote.
(88 KB XLS)

Table S5 A High-Resolution Genetic Map Obtained from Analysis of Eight Sets of RI Lines The columns are the marker name (marker), the genome build of the markers' physical position (genome_build), the chromosome, base pair (bp) position, and genetic position (cM)
(1.1 MB XLS)

## Abbreviations

cM: centimorgan
HS: heterogeneous stock
kb: kilobase
LD: linkage disequilibrium
LINE: long interspersed nuclear element
LOD: log of odds
Mb: megabase
NCBI: National Center for Biotechnology Information
QTL: quantitative trait locus
RI: recombinant inbred
RR: relative risk
SD: standard deviation
SNP: single nucleotide polymorphism
